# Patient Eligibility for Standardized Treatment of the Edentulous Mandible: A Retrospective CBCT-Based Assessment of Mandibular Morphology

**DOI:** 10.3390/jcm8050616

**Published:** 2019-05-07

**Authors:** Walid Aouini, France Lambert, Luc Vrielinck, Bart Vandenberghe

**Affiliations:** 1 Department of Periodontology and Oral Surgery, CHU of Liege, Faculty of Medicine, University of Liege, 4000 Liège, Belgium; aouiniwalid131177@gmail.com (W.A.); france.lambert@gmail.com (F.L.); 2 Department of Oral and Maxillofacial Surgery, St John’s Hospital, 3600 Genk, Belgium; Luc.Vrielinck@zol.be; 3 Advimago, Center for Advanced Oral Imaging, 1050 Brussels, Belgium

**Keywords:** implant, implant-supported prosthetics, standardized prosthetics, mandible morphology, cone-beam computed tomography

## Abstract

The aim of the study was to evaluate the proportion of patients recommended for full-arch mandibular restoration that would be eligible for treatment with a recently developed premanufactured full-arch prosthesis (Trefoil™, Nobel Biocare) based on the morphology of their lower jaw. Anonymized cone beam computed tomography (CBCT) data from 100 partially and fully edentulous patients referred for full-arch mandibular restoration were retrospectively collected from an imaging center database. Using custom-built software, CBCTs of mandibles were registered to a reference CBCT of a patient treated previously with a premanufactured full-arch prosthesis to determine if patients had adequate horizontal width and vertical height for implant placement. Bone height and thickness around simulated implants and distances to the incisive canal were evaluated. Mandibular arch width and semi-automated volume calculations were also performed. Using the system-specific 5.0 mm diameter implants with lengths of 13 and 11.5 mm, 85% and 86% of patients, respectively, were eligible for treatment with the standardized prosthesis. Eligibility was higher for men than women (odds ratio = 3.9, *p* = 0.045) due to increased bone volume. Based on mandibular morphology, our results suggest that the standardized treatment concept could serve a large percentage of patients with edentulous mandibles or failing dentition in the mandible.

## 1. Introduction

Edentulism is a common and debilitating impairment [1,2,3], and mandibular edentulism is particularly troublesome [4]. Current treatments for fully edentulous mandibles include a conventional denture, a removable implant-supported or -retained overdenture stabilized by two locators or a bar, and a fixed implant-supported prosthesis [5,6,7]. When comparing fixed and removable implant-supported dentures, a meta-analysis showed that implant-loss rates were significantly lower for fixed restorations [8], suggesting that fixed implant-supported full-arch prostheses may be a better investment long term. 

The long-term success of a fixed implant-supported prosthesis is highly dependent on the passive fit, with accuracy deviations less than 50–150 µm needed to prevent complications [9]. One of the most popular ways to optimize passive fit is through computer-aided technologies [10], which allows more patients to receive individualized prosthetic frameworks. One advantage of computer-aided design/computer-aided manufacturing (CAD/CAM) concepts is that clinicians can design frameworks that account for prosthodontic constraints and the subsequent reconstruction, allowing the clinician to maximize esthetics and function by modifying biomechanical principles [11]. CAD/CAM frameworks are more accurate than traditional cast frameworks because they reduce errors introduced when manufacturing the prosthesis [9,12]. However, CAD/CAM methodologies are still vulnerable to inaccuracies at the impression stage of the workflow and, to a lesser extent, during the manufacturing process [9,13,14]. Many CAD/CAM procedures still require the use of a provisional prosthesis until the definitive one can be fabricated, requiring multiple post-surgical visits [15]. Further, deviations in implant placement can still occur, even with a customized surgical template [15,16,17]. These deviations could place strains on the framework and bone surrounding the implant and affect the passive fit. While CAD/CAM techniques are clearly successful, they require a substantial investment with respect to both time and cost.

Recently, a standardized prosthetic solution (Trefoil™, Nobel Biocare AB, Gothenburg, Sweden) was introduced [18]. The system consists of three anodized parallel-walled implants featuring a machined collar and conical connection, a standardized single-piece titanium framework and three adaptive fixation mechanisms that compensate for implant placement deviations (horizontal, ±0.4 mm; vertical, ± 0.5 mm; angular, ± 4°). The compensation mechanisms involve a series of articulating discs that allow for limited adjustment in the vertical and horizontal dimensions and multiaxial rotation to achieve a passive fit of the prosthesis. The treatment protocol was also developed with a time-efficient, template-guided clinical workflow and simplified laboratory protocol that can deliver a definitive full-arch mandibular prosthesis on the day of surgery. The passive fit of the Trefoil framework has been shown to be comparable to that of a CAD/CAM superstructure and superior to cast restorations [19].

There are several anatomical features that need to be considered when using the Trefoil concept. After creating a level bone platform, the alveolar crest will need to have sufficient bone (height and width) between the mental foramen to support three implants with a 5.0 mm diameter and lengths of 13 mm or 11.5 mm. Additional anatomical considerations include the inferior dental nerve because the final position of the implants should be mesial to the foramen, avoiding the nerve loop, but should also avoid trauma to the incisive part of the inferior alveolar nerve. Accordingly, the workflow includes a preoperative radiological assessment of mandibular anatomy with an assessment of the vertical dimension of occlusion. Given the standardized nature of this concept, one might assume that this treatment option is limited to patients with a specific mandibular anatomy. 

We hypothesize that the morphological variability of the human mandible is such that a properly designed standardized restorative prosthesis could be used the treat most edentulous patients regardless of gender or age. Thus, the primary aim of this study was to evaluate the proportion of patients who are eligible for treatment with this standardized solution without modification of the standardized framework based on their mandibular morphology. The secondary objective was to estimate the average bone leveling anticipated during the first stage of the surgical protocol while accounting for the minimum bone thickness requirements for implant placement.

## 2. Materials and Methods

### 2.1. Study Design

In this retrospective analysis, cone-beam computed tomography (CBCT) datasets from 100 consecutive patients with partial or fully edentulous mandibles who were recommended for a full-arch mandibular restoration between January 2012 to October 2017 were evaluated for eligibility for the Trefoil standardized treatment concept based on mandibular morphology. Anonymized CBCT datasets were collected from a private imaging clinic in Brussels, Belgium (Advimago, Center for Advanced Oral Imaging). The study included patients of both sexes who had passed cessation of growth. Medical history, smoking, and other exclusion criteria were not considered. 

**Figure 1:**
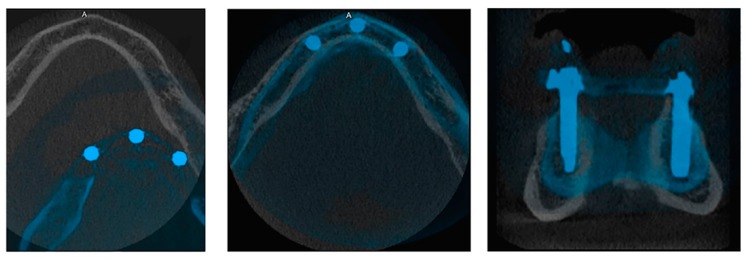
Registration of the master model (MM) and a test mandible (A: anterior). Implants are oriented into the optimal position where they are surrounded by 1 mm of bone.

A customized software platform (MeVisLab, MeVis Medical Solutions AG, Bremen, Germany) was created to allow rigid registration of two CBCT datasets. The platform contained measurement tools for analysis of superimposed datasets. To determine patient eligibility, mandibular CBCT scans were registered with a master model (MM) using automatic rigid registration and voxel resampling. The MM was a CBCT dataset collected 1-year post-surgery from a patient treated with Trefoil at Saint John’s Hospital’s Department of Oral and Maxillofacial Surgery in Genk, Belgium. The framework was seated on 5.0 × 13.0 mm implants. Superimposition of the digital MM was performed manually, so the implant shoulders at the crestal bone platform had at least 1 mm of bone surrounding the circumference of the implant (Figure 1). 

**Figure 2:**
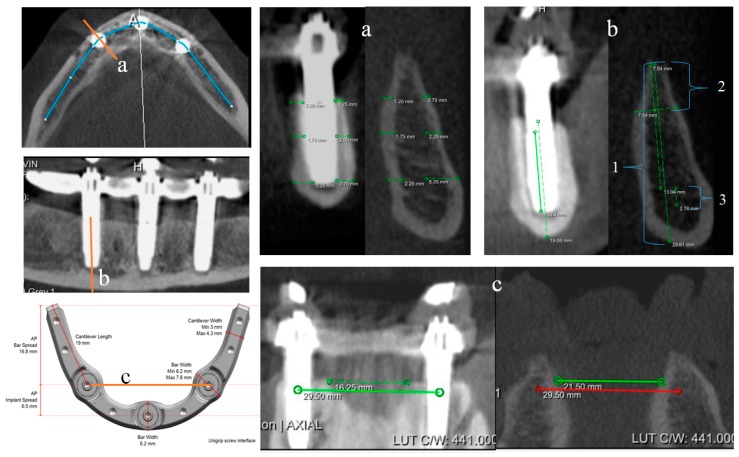
Measurements taken from CBCT images, on the reference model (left) and then transposed and adjusted on the test sample (right) (**a**) Buccal-lingual bone width at apex, middle and platform levels of each implant, (**b**) bone height measurements (1 = total bone height, 2 = bone resection height, 3 = distance from implant apex to incisive canal base), and (**c**) jawbone width measurements.

Linear measurements were collected at three sites, which corresponded to the midsagittal and distal implant positions of the standardized framework. The linear measurements at each implant site included: bone height (total height and resection bone height), buccal and lingual bone width at the implant collar, middle and apex, and distance of the implant apex to the incisive canal base (Figure 2a,b). Mandibular arch widths were measured at 6.5-mm and 23.3-mm posterior to the midsagittal point, which corresponded to the distal implant positions and cantilever terminus, respectively (Figure 2c,d).

Semi-automated volume segmentation was performed on the mandibular jawbone to delimit the area of interest to that supporting the customized framework using region growing. This algorithm selects grey values based on a chosen seed point (e.g., cortical bone) and segments the grey values in the volume representing this type of bone. The threshold for segmenting grey values around this seed point was set visually to segment most of the trabecular and cortical bone. This procedure was followed by a hole-filling algorithm and minor manual adjustments to fill gaps, which represent medullary spaces, to create a filled object. Jawbone volumes of all eligible mandibles were calculated using this filled object. 

Patients were classified as eligible for the standardized treatment if the three simulated implants were placed in a position with alveolar bone around the full circumference and that did not interfere with noble anatomical elements. The measurements of the first 10% of analyzed datasets were repeated after one month to assess intra-observer effects. 

### 2.2. Statistical Analysis

Descriptive statistics were performed with the statistical software SAS version 9.4 (SAS Institute Inc, Cary, NC, USA, https://www.sas.com). Linear measurements (i.e., heights, thicknesses, mandibular width) and semi-automated bone volume calculations are presented as means, standard deviations (SD), and ranges. Intraclass correlation coefficients and paired Student t-tests were used to assess measurement reliability. Results were considered significant at the 5% significance level (*p* < 0.05).

## 3. Results

Among the 100 consecutive patients included, 55% were women and 45% were men. The mean age of patients was 69.8 ± 12.82 years (range 39–91 years); 69% of patients were fully edentulous and 31% were partially edentulous. 

**Figure 3:**
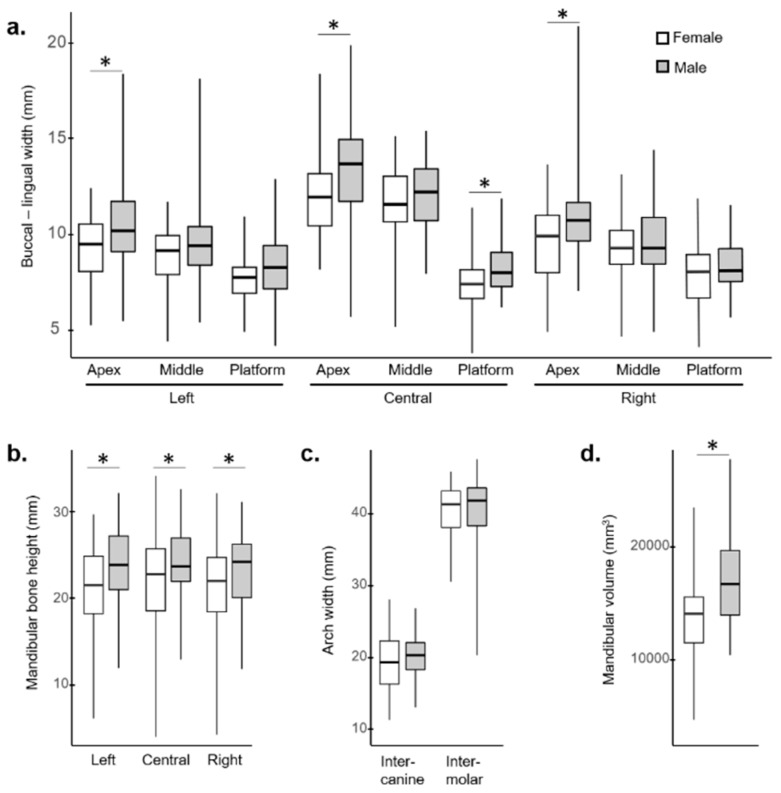
Boxplots depicting the mean measurements of patients who met the eligibility criteria for females (white) and males (shaded). (**a**) Buccal-lingual width at the three implant sites (right, central, left) at three heights (platform, middle, apex), (**b**) total bone height at all three implant sites, arch width at the intercanine (6.5 mm posterior to central implant) and intermolar (16.8 mm posterior to central implant) positions and (**c**) mandibular volume (**d**). Statistical significance (*p* < 0.05) is indicated with an asterisk.

Regarding mandibular morphology measures, there was substantial diversity between patients (Table S1). Total bone heights ranged between 4.10 mm and 34.53 mm. Buccal-lingual thicknesses at the three assessed positions of the implants ranged between 3.88 mm and 21.00 mm. The intercanine distances were between 11.51 mm and 28.25 mm (mean 19.99 mm, *p* > 0.05 between males and females). The intermolar distances were between 20.50 and 47.76 mm (mean 40.74 mm, *p* > 0.05 between males and females), and volumes ranged between 4818 and 27,871 mm^3^ (Figure 3).

Based on mandibular bone morphology, 85% of patients had sufficient bone volume and arch distances to be eligible for treatment with the Trefoil system seated on three 5.0 × 13.0 mm implants. If the framework were placed on the offered 5.0 × 11.5 mm implants, the percentage of eligible patients increased to 86%, and if smaller diameter implants measuring 4.3 × 13 mm (not currently available) were to be used, 89% would be eligible. Eligibility was higher for men than women (odds ratio (OR) = 3.9, *p* = 0.045) due to significantly higher bone volume for men compared with women (17,291 ± 4,409 mm³ vs. 14,340 ± 4,168 mm³, respectively; *p* = 0.0009) (Table 1). Of the 15 patients that did not meet the eligibility criteria, 12 were women, and most were excluded due to insufficient bone height and/or volume (described below). 

For patients with greater inter-foraminal bone volume, there was a significantly higher probability of eligibility (*p* = 0.0002) (Table 2). The main reason for exclusion, assuming a framework seated on three 5.0 × 13.0 mm implants, was due to insufficient bone height and thickness. Seven patients were eliminated because of a horizontal bone deficit, seven were excluded because of insufficient vertical bone, and one was excluded because of a horizontal and vertical bone deficit. Patient age, mandibular arch distances, and degree of edentulousness did not have a significant influence on patient eligibility (all *p* > 0.05, Table 1 and Table 2). Bone volumes of eligible patients ranged between 10,368 and 27,871 mm^3^. Notably, eight of the 15 excluded patients had bone volumes greater than 10,368 mm^3^. This observation was attributed to the shape of the mandible, specifically the symphysis and mandibular horizontal branches, which were high but too thin to receive narrow implants or thick but too short to receive shorter implants.

The surgical protocol for this concept may require resection of alveolar bone to produce a level platform for implant placement. Therefore, the amount of bone that would need to be removed to place the implant with minimal bone volume in all peri-implant dimensions was estimated. The bone resection height ranged from 0.8 to 13.9 mm with a mean of 4.94 ± 2.58 mm, 5.63 ± 2.77 mm, and 5.56 ± 3.00 for the distal right, midsagittal, and distal left sites, respectively. Bone resection height at the midsagittal site was higher compared with the lateral sites (Table 3). This observation was due to the narrowness of the bone crest at this position (Table S3). For all assessed sites, simulated implants were surrounded completely by alveolar bone. The amount of bone around the implants on different sides (buccal and lingual) and at different levels (platform, middle and apex) was greater than or equal to 1 mm in 65.9% of eligible patients for distal right implants, 61.2% of patients for midsagittal implants, and 54.1% of patients for distal left implants (Tables S2–S4). Among the 225 potential implant sites, 42 presented a possible neurological risk (Table 4).

To assess possible intra-observer effects, 10% of patients were re-evaluated one month after measurements were first recorded. Of the 39 datasets re-evaluated, 11 averages were significantly different between the two assessments; however, the overall variability was nonsignificant based on the Student’s t-test (*p* > 0.05). These differences between measurements were likely due to steps in the workflow that required manual adjustments, such as aligning the simulated implants of the master model to the test CBCT data.

## 4. Discussion

In cases of edentulous or soon to be edentulous mandibles, several prosthetic solutions are available to clinicians. Most solutions involve some variation of individualized treatment constructed using either the conventional lost-wax or CAD/CAM techniques [14]. The survival of full-arch implant-supported restorations is high using these individualized solutions, with survival rates of 96.4% after 10 years for conventional techniques [20] and ranging from 92% to 100% with 1–10 years of follow-up for CAD/CAM techniques [21]. However, these customized solutions require several clinical visits and substantial laboratory time, both of which translate to patient time and money. The use of CAD/CAM can potentially reduce the number of visits if the clinician has the appropriate technical and manufacturing capabilities. However, CAD/CAM equipment and software, especially those used for chairside fabrication, require substantial investments in terms of cost and training time [22]. Further, all individualized solutions require the use of a provisional prosthesis, which compounds the time and costs associated with treatment. The standardized restorative concept presented here could eliminate some chair and laboratory time associated with treatment by providing a definitive prosthesis at or near the time of surgery, which would consequently reduce costs. 

The goal of a standardized solution is to give more patients access to implant-supported full-arch prostheses. To realize this goal, a standardized solution must be a feasible treatment option for most edentulous patients. Therefore, in this CBCT-based study, we examined the mandibular morphology of edentulous patients to identify the percentage that would be suitable for this standardized concept. Based on morphology alone this concept could be used to treat up to 86% of patients. The patients that did not fulfill the eligibility criteria, were excluded due to insufficient bone volume at implant sites and jaw shape. Bone volume has a substantial impact on available prosthetic options and should be a consideration in all treatment plans [23]. Our analysis showed that bone volume was a critical factor, leading more men than women to be eligible for this treatment concept. The shape of the jaw, particularly the shape of the symphysis and mandibular horizontal branches, was another factor affecting eligibility. If these structures were too thin, there was not sufficient bone around the potential implant sites. If they were too short, the implants could impinge on the numerous neurovascular structures located in that area [24]. 

With respect to potential surgical parameters, the predicted bone resection was between 3 and 6 mm for 49.4% of eligible patients in the distal right site, 45.9% in the midsagittal site, and 37.6% in the distal left site. In all cases, the proportion of residual bone height after bone planning would be greater than half of the initial value. Previous studies suggest that a safety margin of 2 mm beyond the incisive canal should be preserved to avoid hypoesthesia or dysesthesia [25,26]. The mean incisive canal diameter is estimated to be 1.8 mm [27]. Therefore, the minimum safety distance from the implant apex to the base of the incisive canal would be 3.8 mm. Given this criterion, between 9.4% and 22.4% of eligible patients would have some risk of the implants interfering with the nerve. However, this concept requires a minimum bone height to be eligible for treatment, and the degree of bone leveling depends primarily on the occlusion of the prosthesis. Thus, for many patients, more bone height is preserved before implants are placed. Furthermore, if an individual case presents with a surgical risk, implants could be placed through the incisive canal, while avoiding compression. Given the importance of jaw volume and shape as well as bone height, a clinical evaluation of jaw size, jaw relations, intermaxillary distance, occlusal relation, and the condition of the opposing dentition is paramount with this standardized concept.

There are several limitations to this analysis. First, the eligibility was calculated based on bone morphology and does not account for patients who would be medically unfit for an oral surgery procedure (e.g., those with inadequate mouth opening, poor occlusion, insufficient final torque, poor bone quality). Second, the MM was based on the CBCT data of one patient rather than the average of several existing patients. Third, we modeled primarily for the 5.0 mm diameter, 13.0 mm length implants recommended by the manufacturer. More patients could potentially be eligible if smaller implants were used. Fourth, because the sample population was anonymized, we could not account for the ethnicity of the patients, which could influence the eligibility results. However, Brussels is one of the most diverse cities in the world according to the International Organization for Migration, with 62% of residents being foreign-born, primarily of Turkish and African descent [28]. 

Within the limits of this study, we found that this standardized implant-supported full-arch prosthesis could treat as many as 86% of the population based on mandibular morphology and the size of the treatment-specific implants. Given the standardized framework, lack of a provisional prosthesis, and a reduced number of clinical visits, this treatment protocol could be an effective solution that can feasibly treat a large number of patients. Ongoing and future clinical studies will determine the effectiveness of this device long term.

## Figures and Tables

**Table 1:** Patient eligibility according to sex and type of edentulousness.

		Excluded		Eligible			
Variable	Categories	Total *n*	*n* (%)	Total *n*	*n* (%)	Odds Ratio	*p*-Value
Sex		15		85			0.045
	Female		12 (80.0)		43 (50.6)	1.00	
	Male		3 (20.0)		42 (49.4)	3.91	
Edentulousness		15		85			0.32
	Totally edentulous		12 (80.0)		57 (67.1)	1.00	
	Residual teeth		3 (20.0)		28 (32.9)	1.97	

**Table 2:** Influence of age, mandibular arch, and volume on patient eligibility.

Variable	*n*	Mean	SD	Min	Q1	Median	Q3	Max	*p*-Value
Age (years)									0.26
Excluded	15	73.27	13.750	50.00	61.00	79.00	85.00	90.00	
Eligible	85	69.19	12.634	39.00	62.00	71.00	78.00	91.00	
Intercanine distance (mm)									0.74
Excluded	15	20.28	4.014	11.51	17.25	21.30	23.00	25.90	
Eligible	85	19.94	3.491	13.06	17.53	19.51	22.01	28.25	
Intermolar distance (mm)									0.17
Excluded	15	42.06	2.587	36.44	41.06	42.11	44.25	45.00	
Eligible	85	40.50	4.244	20.50	38.26	41.50	43.35	47.76	
Volume (mm^3^)									0.0002
Excluded	15	10428	3447.5	4817.5	8046.3	10676	11619	16314	
Eligible	85	16593	4020.3	10368	13769	15504	19278	27871	

Abbreviations: Q1, first quartile; Q3, third quartile; SD, standard deviation.

**Table 3:** Bone resection at each implant site for eligible patients and the master model.

Bone resection Value (mm)	Distal Right	Midsagittal	Distal Left
<3, *n* (%)	17 (20.0)	14 (16.5)	18 (21.2)
≥3 and <6, *n* (%)	42 (49.4)	39 (45.9)	32 (37.6)
≥6 and <9, *n* (%)	20 (23.5)	19 (22.4)	23 (27.1)
≥9, *n* (%)	6 (7.1)	13 (15.3)	12 (14.1)
Mean ± SD	4.94 ± 2.58	5.63 ± 2.77	5.56 ± 3.00
Range (min–max)	0.8–12.3	0.8–13.9	0.8–13.8
Master model	8.56	7.89	10.5

Abbreviations: SD, standard deviation.

**Table 4:** Distance from implant apex to the incisive canal base.

Distance from Implant Apex to the Incisive Canal Base (mm)	Neurological Risk	Right Site # (%)	Midsagittal Site # (%)	Left Site # (%)
0–3.8 mm	Yes (Implant apex may compress the nerve)	15 (17.6)	19 (22.4)	8 (9.4)
>3.8	No (Implant apex away from the nerve)	2 (2.4)	4 (4.7)	5 (5.9)
<0	No (Implant crosses the incisive canal)	68 (80.0)	62 (72.9)	72 (84.7)
Mean ± SD		−2.96 ± 3.31	−3.06 ± 3.63	−3.51 ± 3.33
Range		−10.5–7.3	−10.0–6.8	−9.8–7.8
Master model		−2.26	−2.00	−2.25

## Supplementary Materials

The following are available online at https://www.mdpi.com/2077-0383/8/5/616/s1, Table S1: Mandibular measurements, Table S2: Amount of bone around the right implant on the buccal and lingual sides at the platform, middle, and apical levels, Table S3: Amount of bone around the middle implant on the buccal and lingual sides at the platform, middle and apical levels, Table S4: Amount of bone around the left implant on the buccal and lingual sides at the platform, middle and apical levels.
