# Supplementary material for: Patient Eligibility for Standardized Treatment of the Edentulous Mandible: A Retrospective CBCT-Based Assessment of Mandibular Morphology
Source: J Clin Med. 2019 May 7;8(5):616. doi: 10.3390/jcm8050616 (PMC6572614; doi:10.3390/jcm8050616)
Supplement: Supplementary file 1 [file jcm-08-00616-s001.pdf]

Journal of Clinical Medicine

**Supplementary Materials**

**Table S1.** Mandibular measurements.

**Table S2.** Amount of bone around the right implant on the buccal and lingual sides at the platform, middle, and apical levels.

**Table S3.** Amount of bone around the middle implant on the buccal and lingual sides at the platform, middle and apical levels

**Table S4.** Amount of bone around the left implant on the buccal and lingual sides at the platform, middle and apical levels

**Table S1.** Mandibular measurements.

| Variable | Mean | SD | Min | Q1 | Median | Q3 | Max |
|---|---|---|---|---|---|---|---|
| Intercanine distance (mm) | 19.99 | 3.554 | 11.51 | 17.53 | 20.24 | 22.38 | 28.25 |
| Intermolar distance (mm) | 40.74 | 4.067 | 20.50 | 38.48 | 41.54 | 43.68 | 47.76 |
| Mandibular volume (mm3) | 15668 | 4504.2 | 4817.5 | 12465 | 15048 | 18920 | 27871 |
| Assessment of right site (mm) | | | | | | | |
| Total bone height | 22.42 | 5.309 | 4.37 | 19.75 | 23.20 | 25.68 | 32.50 |
| Bone thickness at implant platform | 8.30 | 1.522 | 4.21 | 7.50 | 8.15 | 9.25 | 11.98 |
| Bone thickness at implant middle | 9.47 | 1.810 | 4.75 | 8.54 | 9.38 | 10.75 | 14.52 |
| Bone thickness at implant apex | 10.41 | 2.327 | 5.00 | 9.02 | 10.56 | 11.58 | 21.00 |
| Assessment of midsagittal site (mm) | | | | | | | |
| Total bone height | 23.25 | 4.891 | 4.10 | 20.53 | 23.24 | 26.56 | 34.53 |
| Bone thickness at implant platform | 7.97 | 1.481 | 3.88 | 7.00 | 7.76 | 8.75 | 11.97 |
| Bone thickness at implant middle | 11.94 | 1.812 | 5.27 | 10.77 | 12.01 | 13.26 | 15.51 |
| Bone thickness at implant apex | 12.81 | 2.556 | 5.79 | 11.05 | 12.77 | 14.34 | 20.00 |
| Assessment of left site (mm) | | | | | | | |
| Total bone height | 22.47 | 5.200 | 6.25 | 19.81 | 23.00 | 26.16 | 32.52 |
| Bone thickness at implant platform | 8.12 | 1.629 | 4.27 | 7.25 | 8.00 | 9.00 | 13.00 |
| Bone thickness at implant middle | 9.23 | 1.877 | 4.50 | 8.26 | 9.25 | 10.30 | 18.25 |
| Bone thickness at implant apex | 9.89 | 2.091 | 5.35 | 8.53 | 10.03 | 11.04 | 18.50 |

**Table S2.** Amount of bone around the right implant on the buccal and lingual sides at the platform, middle, and apical levels.

| | Buccal side | | | Lingual side | | |
|---|---|---|---|---|---|---|
| **Bone thickness (mm)** | **Platform level** | **Middle level** | **Apical level** | **Platform level** | **Middle level** | **Apical level** |
| <0, N (%) | 0 (0.0) | 0 (0.0) | 0 (0.0) | 0 (0.0) | 0 (0.0) | 0 (0.0) |
| ≥ 0 and <1, N (%) | 29 (34.1) | 9 (10.6) | 4 (4.7) | 17 (20.0) | 6 (7.1) | 7 (8.2) |
| ≥ 1, N (%) | 56 (65.9) | 76 (89.4) | 81 (95.3) | 68 (80.0) | 79 (92.9) | 78 (91.8) |
| Mean | 1.46 | 2.09 | 2.98 | 1.99 | 2.53 | 2.64 |
| SD | 0.93 | 1.01 | 1.42 | 1.10 | 1.10 | 1.48 |
| Min | 0.0 | 0.0 | 0.0 | 0.0 | 0.0 | 0.0 |
| Max | 4.0 | 4.6 | 8.3 | 4.5 | 5.6 | 7.8 |

Abbreviations: SD, standard deviation

**Table S3.** Amount of bone around the middle implant on the buccal and lingual sides at the platform, middle and apical levels

| | Buccal side | | | Lingual side | | |
|---|---|---|---|---|---|---|
| **Bone thickness (mm)** | **Platform level** | **Middle level** | **Apical level** | **Platform level** | **Middle level** | **Apical level** |
| <0, N (%) | 0 (0.0) | 0 (0.0) | 0 (0.0) | 0 (0.0) | 0 (0.0) | 0 (0.0) |
| ≥ 0 and <1, N (%) | 33 (38.8) | 4 (4.7) | 4 (4.7) | 25 (29.4) | 0 (0.0) | 2 (2.4) |
| ≥ 1, N (%) | 52 (61.2) | 81 (95.3) | 81 (95.3) | 60 (70.6) | 85 (100.0) | 83 (97.6) |
| Mean | 1.31 | 2.31 | 3.41 | 1.71 | 4.83 | 4.63 |
| SD | 0.80 | 1.06 | 1.48 | 1.24 | 1.40 | 1.95 |
| Min | 0.0 | 0.0 | 0.0 | 0.0 | 1.5 | 0.0 |
| Max | 4.4 | 4.6 | 8.0 | 5.8 | 8.5 | 8.9 |

Abbreviations: SD, standard deviatio

**Table S4.** Amount of bone around the left implant on the buccal and lingual sides at the platform, middle and apical levels

| | **Buccal side** | | | **Lingual side** | | |
|---|---|---|---|---|---|---|
| **Bone thickness (mm)** | **Platform level** | **Middle level** | **Apical level** | **Platform level** | **Middle level** | **Apical level** |
| <0, N (%) | 0 (0.0) | 0 (0.0) | 0 (0.0) | 0 (0.0) | 1 (1.2) | 0 (0.0) |
| ≥ 0 and <1, N (%) | 9 (10.6) | 5 (5.9) | 10 (11.8) | 39 (45.9) | 11 (12.9) | 12 (14.1) |
| ≥ 1, N (%) | 76 (89.4) | 80 (94.1) | 75 (88.2) | 46 (54.1) | 73 (85.9) | 73 (85.9) |
| Mean | 2.14 | 2.42 | 2.98 | 1.15 | 1.96 | 2.08 |
| SD | 1.20 | 1.09 | 1.56 | 1.08 | 1.17 | 1.04 |
| Min | 0.0 | 0.0 | 0.0 | 0.0 | 1.0 | 0.0 |
| Max | 5.9 | 6.0 | 8.0 | 6.8 | 7.3 | 5.5 |

Abbreviations: SD, standard deviation
